# Supplementary material for: Conditional chemoconnectomics (cCCTomics) as a strategy for efficient and conditional targeting of chemical transmission
Source: eLife. 2024 Apr 30;12:RP91927. doi: 10.7554/eLife.91927 (PMC11060718; doi:10.7554/eLife.91927)
Supplement: Figure 3—source data 3. [file elife-91927-fig3-data3.zip › Figure 3 source data3 part 1.zip!/Figure 3-Source data 3-part1/1063f3-1-CRISPResso_Report_kboyr13q.zip!/CRISPResso_on_1063f3_4/1a.Read_barplot.pdf]

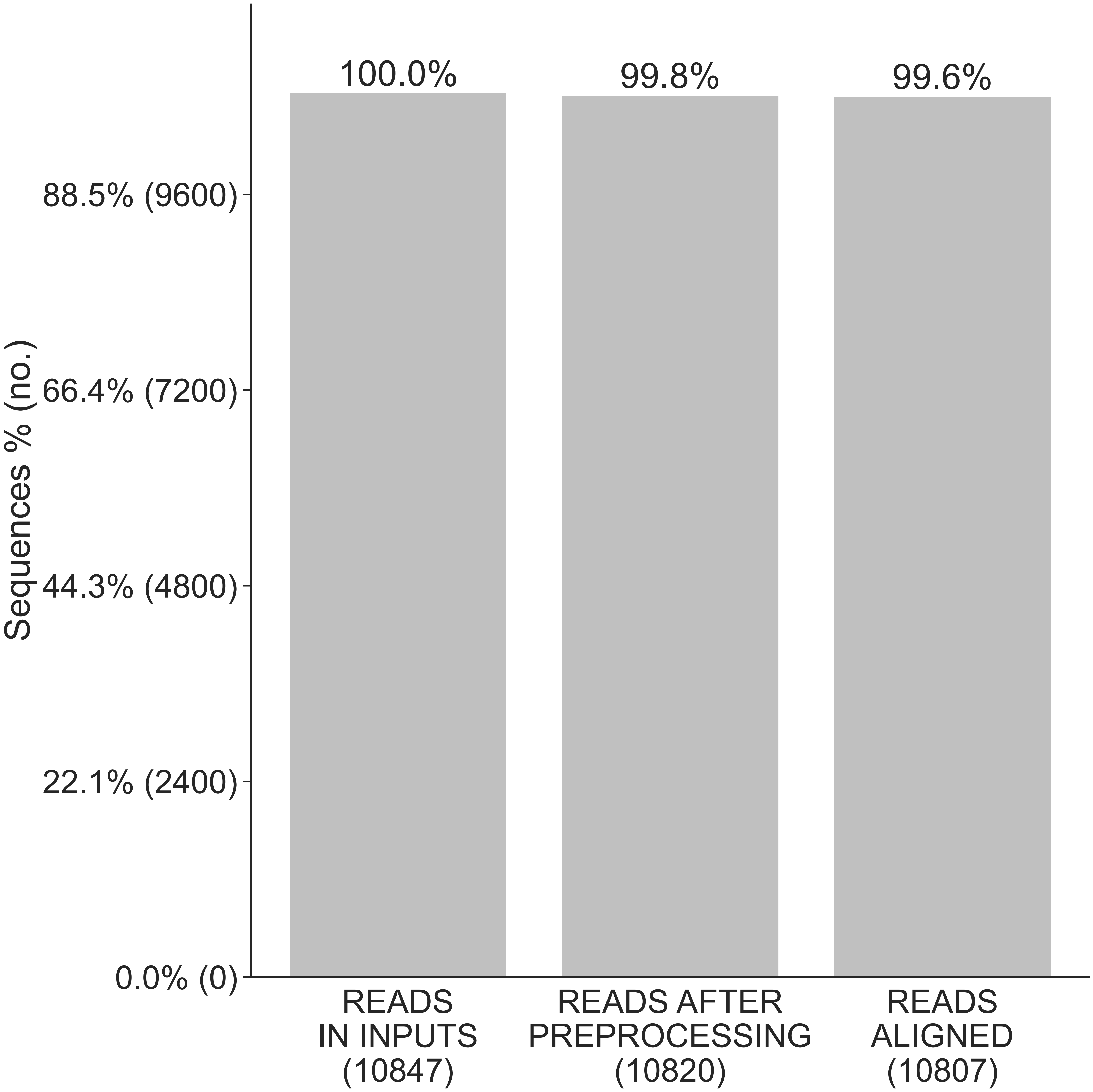
Sequences % (no.)
88.5% (9600)
66.4% (7200)
44.3% (4800)
22.1% (2400)
0.0% (0)
100.0%
99.8%
99.6%
READS IN INPUTS (10847)
READS AFTER PREPROCESSING (10820)
READS ALIGNED (10807)
